# Efficacy and safety of combination of frankincense and botulinum toxin in the treatment of blepharospasm: a protocol for a single-centre, open-label, randomized, controlled trial

**DOI:** 10.3389/fneur.2025.1693914

**Published:** 2025-12-01

**Authors:** Shiyuan Gong, Yuhan Luo, Jiana Zhang, Feiwen Huang, Zhengkun Yang, Yue Zhang, Zilin Ou, Zhicong Yan, Weixi Zhang, Qian Zhou, Gang Liu

**Affiliations:** 1 Department of Neurology, The First Affiliated Hospital, Sun Yat-sen University, Guangdong Provincial Key Laboratory of Diagnosis and Treatment of Major Neurological Diseases, National Key Clinical Department and Key Discipline of Neurology, Guangzhou, China; 2 Department of Medical Statistics, Clinical Trials Unit, The First Affiliated Hospital of Sun Yat-sen University, Guangzhou, China

**Keywords:** blepharospasm, botulinum toxins, clinical trial protocol, frankincense, randomized controlled trial

## Abstract

**Introduction:**

Blepharospasm (BSP) is a functionally disabling disease with a marked impact on the quality of life of patients. Botulinum toxin (BoNT) injections have been recommended as first-line therapy for BSP. However, the clinical benefits of BoNT are temporary with only about 8–10 weeks duration of benefit in most patients with BSP. Per previous case reports, regular use of topical frankincense essential oil (FEO) can achieve significant symptom relief and can decrease the frequency of BoNT injections. This trial will explore whether BoNT combined with regular application of FEO can improve clinical outcomes in patients with BSP.

**Methods and analysis:**

This protocol describes an open-label, randomized controlled trial to be undertaken to evaluate daily topical application of FEO and coconut oil (CO) in patients with BSP. The goal is to enroll 32 patients with BSP who have received immediate BoNT injection in each treatment arm. Only patients who have previously received less than 12 weeks of positive benefits from BoNT therapy will be enrolled. The primary outcome will be the duration of symptom improvement after the intervention with BoNT combined with FEO or CO within the 24-week follow-up period. Symptom improvement is defined as a decrease of one point or more in the Jankovic Rating Scale severity score compared to baseline in patients with BSP. Secondary outcomes will consist of changes in BSP symptom severity, disability, and quality of life from baseline to each time point after the intervention. Safety analysis will be based on the presence of localized skin allergic reactions and adverse events. Outcomes will be assessed at baseline and at weeks 2, 4, 8, 12, 16, 20, and 24 after the therapy begins.

**Discussion:**

This study will provide evidence that FEO therapy is a promising non-invasive therapy that can be easily combined with BoNT injections to improve clinical outcomes in patients with BSP.

**Clinical trial registration:**

https://www.chictr.org.cn/, identifier ChiCTR2400091987.

## 1 Introduction

Blepharospasm (BSP), characterized by involuntary bilateral orbicularis oculi muscle spasms, is the most common type of focal dystonia in Asia (1–3). BSP significantly affects the quality of life of patients through motor and non-motor manifestations (3–6). Currently, BSP treatment remains purely symptomatic, owing to its unknown etiology. Oral medications, such as benzodiazepines, anticholinergics, antimuscarinics, and dopaminergic medications, exert temporary benefits, but these vary from patient to patient, generally lack long-term effectiveness, and result in significant side effects (7). Surgical myectomy is invasive and is usually recommended for patients who do not respond to medication (7). Botulinum toxin (BoNT) injections are widely regarded as the gold standard therapy for patients with BSP; however, the clinical benefits of BoNT injections begin to wear off after 8–10 weeks in most patients (8–11). As a result, most patients must receive reinjections after 12 weeks or 4–5 times per year (7). Therefore, there is an urgent need to develop novel adjunct therapies.

Many studies have shown the transient therapeutic benefits of non-invasive brain stimulation (NIBS), such as repetitive transcranial magnetic stimulation (rTMS) and transcranial direct current stimulation (8, 12–15). Recently, Shukla et al. (8) examined whether combined treatment with rTMS and BoNT injections could improve the clinical outcomes in BSP. They recruited 12 patients with BSP and randomly assigned them to real rTMS or sham rTMS treatment groups. The intervention was conducted 1 month after BoNT and lasted for a 2-week course. The combined approach to the anterior cingulate cortex (ACC) with a double-cone coil and BoNT could significantly improve motor symptoms, quality of life, and social life at 2 weeks compared with BoNT alone. However, these benefits were no longer evident at 4 weeks after completion of rTMS, suggesting that rTMS may offer an adjunctive benefit in BSP. Although the findings of Shukla et al. (8) are encouraging, the application of NIBS in BSP treatment faces several significant challenges. First, the sample size was small, and the benefits of rTMS appear to be short-term. Second, although studies have reported the ACC, primary motor cortex, and dorsal premotor cortex as potential stimulation targets for BSP treatment, the optimal stimulation target regions remain unclear (12). Finally, NIBS treatment may be inaccessible to some patients because of the high equipment requirements. Therefore, there is a need to explore alternative adjunctive therapies that are non-invasive and easily integrated into standard BoNT regimens. A recent case report described two patients with BSP who used topical frankincense essential oil (FEO) regularly (16). Both patients showed significant symptomatic improvement and a reduced frequency of BoNT type A (BoNT-A) injections. Patient 1 experienced an extension in the injection interval from every 5–8 months to 11 + months and eventually stopped BoNT treatment after starting FEO. The frequency of BoNT injections declined from every 3–4 months to 8 months in patient 2. FEO extracted from Boswellia tree resin has a long history of use in traditional medicine (17). FEO has potential therapeutic effects and treats neurological diseases, such as Parkinson’s disease, Alzheimer’s disease, and multiple sclerosis, through anti-inflammatory and antioxidant effect (18–20). Recent clinical evidence suggests that oxidative stress may contribute to the pathophysiology of dystonia (21). This finding indicates that antioxidant compounds, such as Boswellia extracts, could be relevant in this context. Despite these indications, the application of FEO in the treatment of BSP has not been extensively evaluated. Therefore, it is necessary to explore whether topical FEO may serve as an adjunct treatment to improve clinical outcomes in patients with BSP, so as to exploit its inexpensive and non-invasive nature.

We plan to collect data using this novel combination treatment and assess the efficacy and safety of combining topical FEO with BoNT in patients with BSP.

## 2 Methods and analysis

### 2.1 Trial design

We will conduct a single-centre, open-label, randomised controlled trial. Patients diagnosed with BSP receiving BoNT treatment at our centre will be approached. The protocol follows the Standard Protocol Items: Recommendations for Interventional Trials guidelines (22). Outcomes, including symptom severity, disability, and quality of life, will be evaluated at baseline and at weeks 2, 4, 8, 12, 16, 20, and 24 post intervention. Patients will be asked to complete a self-report questionnaire to document their response to BoNT and the duration of clinical benefits after BoNT treatment. We will recruit only those patients who have received positive benefits from BoNT for less than 12 weeks. We plan to enroll 64 patients and randomly (1:1) assign them to the experimental group (topical FEO combined with BoNT-A injection) or control group [topical coconut oil (CO) combined with BoNT-A injection]. CO will be used as the control because FEO must be diluted 1:1 with CO as the carrier oil (16).

### 2.2 Study population

All patients who meet the following inclusion criteria will be included: (1) age 18–70 years; (2) a diagnosis of BSP established according to the published criteria by a senior neurologist (2); (3) a Jankovic Rating Scale (JRS) frequency sub-score of ≥3; (4) documented history of at least three subsequent injection cycles or prospective documentation of one injection cycle of BoNT with less than 12 weeks duration of positive benefits with BoNT by a BoNT-experienced physician (23); (5) written consent obtained from participants. The exclusion criteria include: (1) family history or diagnosis of hereditary dystonia, acquired dystonia, tardive dyskinesia, or functional movement disorders; (2) presence of acute intracranial disease (e.g., stroke, intracranial infection) or history of psychiatric disorders, dementia, epilepsy, multiple sclerosis, tic disorder, Parkinson’s disease, severe traumatic brain injury, or other neurological diseases; (3) significant cognitive impairment; (4) exposure to neuroleptics; (5) contraindication to magnetic resonance imaging (MRI).

### 2.3 Objectives and endpoints

Primary objectives: The study primarily aims to evaluate the clinical effects of topical FEO combined with BoNT injections in patients with BSP. Specifically, we will compare the topical FEO group with the CO group in terms of the duration of symptom improvement, quality of life, and self-rating of response to efficacy during a 24-week follow-up. Symptom improvement is defined as a decrease of one point or more in the JRS severity in the JRS severity score relative to baseline in patients with BSP (24). We hypothesized that, unlike the placebo group, topical FEO combined with BoNT will significantly prolong the duration of symptom improvement, improve patient rating of quality of life, and self-rating of response to efficacy, with acceptable safety at the 24-week follow-up.

The primary endpoint is the duration of symptom improvement over 24 weeks, as defined above.

The secondary endpoint of the study is the percentage change from baseline to different time points in the physician rating of the BSP. This involves motor symptom severity, measured by the JRS (25), the Blepharospasm Severity Rating Scale (BSRS) (26), and the Burke Fahn Marsden’s Dystonia Rating Scales (BFMDRS) (27), and quality of life, assessed by the Blepharospasm Disability Index (BSDI) (25), the Short Form-36 Health Survey (SF-36) (28), and the Cranio-cervical Dystonia Questionnaire (CDQ-24) (29). In addition, patient subjective ratings via the Patient Evaluation of Global Response (PEGR) and the incidence of adverse events will be included (30).

### 2.4 Data measurements

**Figure 1:**
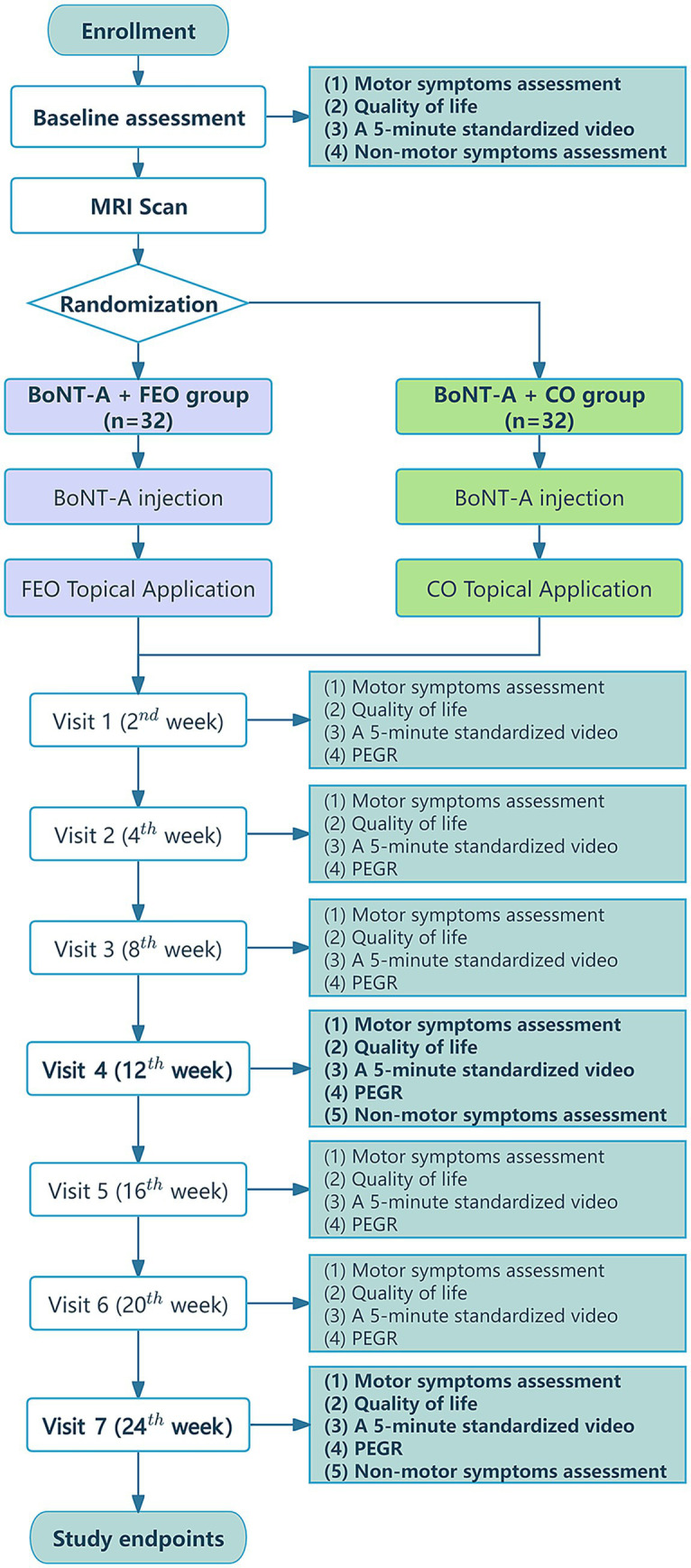
Clinical trial flow diagram. BoNT-A, botulinum toxin type A; CO, coconut oil; FEO, frankincense essential oil; MRI, magnetic resonance imaging; PEGR, Patient Evaluation of Global Response.

All subjects will undergo MRI before the clinical intervention. Demographics, clinical characteristics, and severity of BSP will be recorded using blepharospasm severity scales. The severity of BSP, quality of life, and self-rating of response to efficacy will be evaluated in each patient at baseline and at weeks 2, 4, 8, 12, 16, 20, and 24 after therapy. Moreover, a 5-min standardised video recording of the facial area will be conducted to assess and document the blink rate and duration of sustained eyelid closure (26). In addition, all subjects will be evaluated with non-motor symptom scales at baseline, at weeks 12 and 24 weeks after the intervention, using the Mini-Mental State Examination (MMSE) (31), Hamilton Rating Scale for Anxiety (HAMA) (32), Hamilton Rating Scale for Depression (HAMD) (33), Yale-Brown Obsessive-Compulsive Scale (Y-BOCS) (34), and the Pittsburgh Sleep Quality Index (PSQI) (35). All evaluations applied in the study will be performed at each visit by two evaluators who will be blinded to the clinical information and group assignments of the participants. This will safeguard the objectivity of the evaluation process and ensure the consistency and validity of the assessment results. Figure 1 illustrates the flowchart of the clinical trial procedure, and Table 1 presents an overview of the study.

**Table 1:** Overview of the study.

Item	Description
Study title	Efficacy and safety of combination of frankincense and botulinum toxin in the treatment of blepharospasm: a protocol for a single-centre, open-label, randomized, controlled trial
Study design	A single-centre, open-label, randomized, controlled trial will be conducted in 64 eligible BSP patients, and they will be randomly assigned (1:1) into the experimental group (topical FEO combined with BoNT) and control group (topical CO combined with BoNT) for the 24-week follow-up.
Primary endpoint	The symptom improvement over a 24-week follow-up period.
Secondary endpoints	Intervals between the last and the next BoNT treatments; Improvement rates in motor symptom severity (JRS, BSRS, and BFMDRS scores); Improvement rates in the quality of life (BSDI, SF-36, and CDQ-24 scores); Patient subjective efficacy evaluation (PEGR scores); Incidence of adverse events.
Research intervention(s)/Investigational agent(s)	Daily application topical FEO the day after BoNT injection in the experimental group. Daily topical application of CO the day after BoNT injection in the control group. Outcomes will be collected at baseline and different follow-up time points. BoNT: Hengli. FEO/CO: Aromatics International.
Study population	Patients aged 18 to 70 years whose efficacy duration of BoNT injection was less than 12 weeks.
Sample size	64
Study duration for individual participants	24 weeks

BFMDRS, Burke Fahn Marsden’s Dystonia Rating Scales; BoNT, botulinum toxin; BSDI, Blepharospasm Disability Index; BSP, blepharospasm; BSRS, Blepharospasm Severity Rating Scale; CDQ-24, Cranio-cervical Dystonia Questionnaire; CO, coconut oil; FEO, frankincense essential oil; JRS, Jankovic Rating Scale; SF-36, Short Form-36 Health Survey; PEGR, Patient Evaluation of Global Response.

### 2.5 Safety evaluation

Based on previous studies, the oral use of frankincense occasionally or rarely causes adverse reactions such as eczema, nausea, aggravation of arthralgia, and gastrointestinal symptoms (20, 36, 37). Topical application of FEO has fewer side effects, predominantly in the form of localized skin allergic reactions (38). All adverse events, including their description, duration, severity, and required treatment, will be documented and treated with care. Any serious adverse events will be immediately reported to the chief investigator and ethics committee of the First Affiliated Hospital of Sun Yat–sen University, and the ethics committee will ultimately determine the termination of the study.

### 2.6 Sample size

The sample size was calculated using the two-sample t-test method in PASS 15 software (NCSS, LLC, Kaysville, Utah, USA), with a two-sided significance level of 0.05 and a power of 0.8. Based on preliminary clinical observations obtained in our center, topical FEO alone was effective in approximately 50% of patients with BSP. This finding, combined with a conservative estimate of efficacy, prompted a reassessment of the required sample size to ensure adequate statistical power. The revision was implemented after trial initiation but prior to completion of participant recruitment, and constitutes an important protocol modification based on internally generated data. To determine the mean and standard deviation (SD) needed for the sample size calculation, we retrospectively analysed data from 61 patients with BSP at our movement disorders clinic who had received standard BoNT-A treatment and met the study’s inclusion criteria. After receiving their last three consecutive BoNT-A injections, these patients experienced clinical benefits for less than 3 months each time, demonstrating consistently unsatisfactory therapeutic effects. The mean duration of clinical benefits was 73.99 days (SD = 14.28). Given that the control group will receive a placebo, there may have been greater variability in the therapeutic effects due to individual response and disease progression; therefore, we decided to round the mean value to 75 days and the SD to 15 (i.e., 75.0 ± 15.0) to ensure robustness in the calculation. In this study, we hypothesized that combining BoNT-A injection with topical FEO would extend the effective duration of symptom relief by 30 days to 105.0 ± 15.0 days. Given an expected effective rate of only 50%, we estimated the mean duration of benefit in the experimental group as 90.0 ± 18.5 days, reflecting a weighted average between responders and non-responders. The resulting sample size calculation indicated N1 = N2 = 25, meaning that 25 participants are required for each group. Considering the participant loss to follow-up, we determined that each group would recruit 32 participants. A professional statistician verified the sample size calculation method. The amended protocol was reviewed and approved by the Ethics Committee of the First Affiliated Hospital of Sun Yat–sen University.

### 2.7 Randomization and recruitment

The randomization procedure will be managed and documented entirely by an independent statistician who is not engaged in the implementation of the study. Sixty-four random numbers were generated using IBM SPSS Statistics (v.25.0; IBM Corp., Armonk, NY). These random numbers will then be allocated equally to two groups using the visual split-box function in SPSS. Another designated blinded investigator is responsible for assigning participants to different interventions. To ensure allocation concealment, other investigators, including participant recruiters and evaluators, will remain unaware of the randomization results until the study is fully completed. Using this approach, the study will effectively accomplish randomization and allocation concealment, thereby minimizing selection bias and ensuring the validity and reliability of the results.

The intended recruitment period is approximately 2 years and 2 months, from 28 December 2024, to 28 February 2027. The participants will be free to withdraw from the study at any time. The reasons for withdrawal will be documented. If the reason is associated with adverse events, it will be followed up until the issue is completely resolved. Participants who have received other treatments or participated in another program related to BSP therapy during the trial will be considered dropouts.

### 2.8 Intervention administration

#### 2.8.1 Topical FEO combined with BoNT group

**Figure 2:**
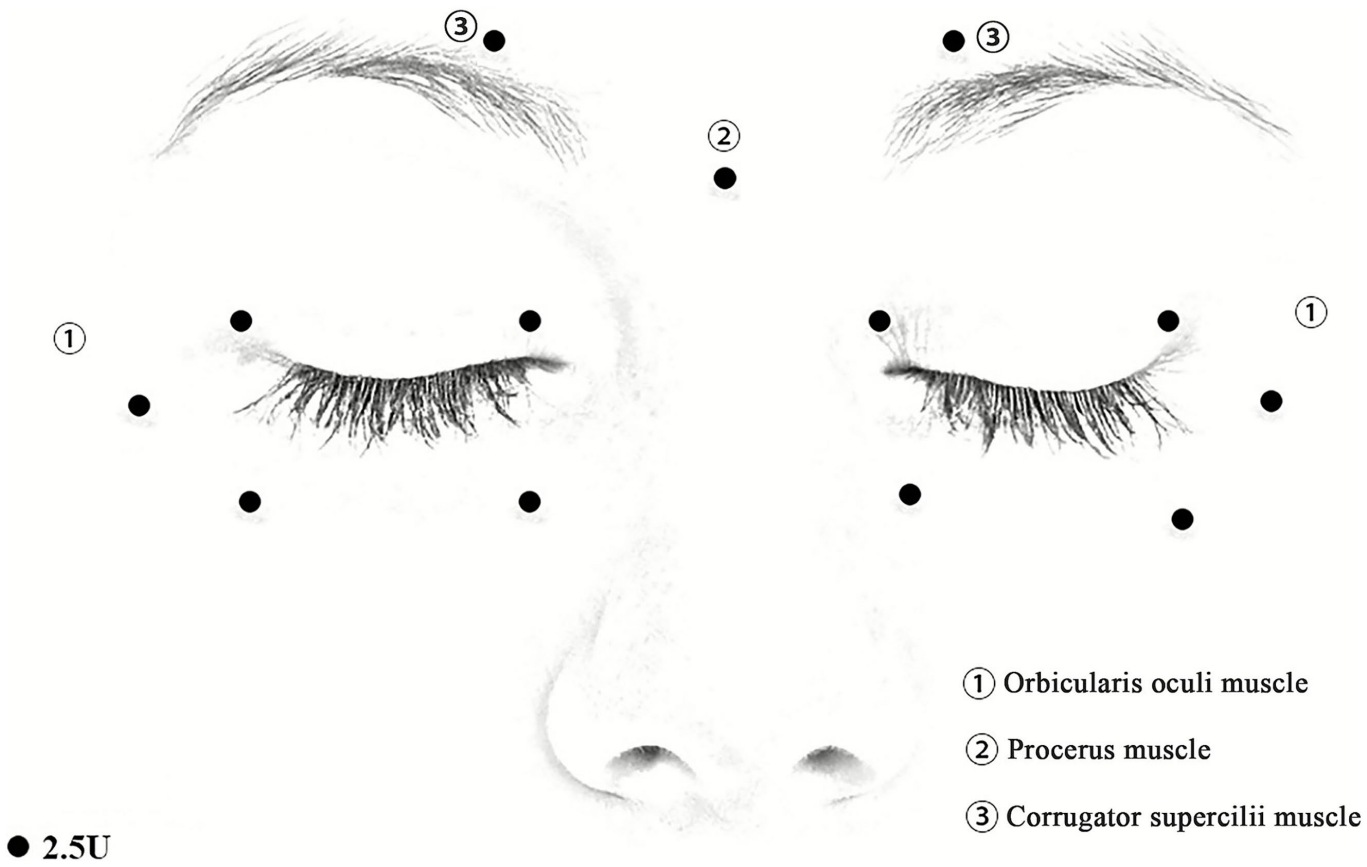
Schematic diagram of BoNT-A injection sites. (1) Injection sites on the orbicularis oculis. (2) Injection sites on the procerus muscle. (3) Injection sites on the corrugator supercilii muscles. The doses of BoNT-A: 2.5 U/dot.

Participants in the experimental group will receive injections of BoNT-A (Hengli, Lanzhou Biological Products Institute, Lanzhou, China; 100 U, diluted in 2 mL saline), using 1 mL syringes and manual targeting techniques. BoNT-A will be routinely administered to the orbicularis oculi muscles at a total of 10 points (5 points on each side). These points encompass the medial and lateral one-third of both the upper and lower eyelids, 2–3 mm from the eyelid margin, and the temporal orbicularis oculi muscle, located 5 mm from the lateral canthus. The corrugator supercilii muscles will receive two injections (one point on each side) located above the inner side of the eyebrows. Additionally, BoNT-A will be injected into the procerus muscle at a central point between the eyebrows. Each point will receive injection of 2.5 U BoNT-A, and the total BoNT-A dosage will be approximately 32.5 U per participant (Figure 2). The injection site and dosage will be adjusted slightly based on the patient’s condition. The next day, participants in the experimental group will begin applying the FEO mixture to the bilateral upper and lower eyelids twice daily, using 0.25 mL of the mixture per application, 0.125 mL for each side. The application will be performed after cleaning the face, and participants will be instructed to gently massage the oil mixture into the skin and rest with their eyes closed to allow absorption.

#### 2.8.2 Topical CO combined with BoNT group

In the control group, the method of injecting BoNT-A and CO will be consistent with that of the experimental group, including site, dosage, and frequency. Participants will be provided with detailed instructions and training on the correct application of the oils, and their adherence to the intervention protocol will be monitored through regular follow-ups. FEO and CO used in the trial are supplied by Aromatics International.^1^

### 2.9 Trial assessment and follow-up

Full eligibility criteria will be assessed and confirmed before randomization. Further assessments should be conducted at multiple time points. An overview of the data collection process is provided in Table 2. Data will be collected from all participants until they complete the 24-week follow-up period or discontinue the study.

**Table 2:** Time and events.

Description	Screening	Day 0	2nd week	4th week	8th week	12th week	16th week	20th week	24th week
Consent	**√**								
Neurological exam	**√**								
Medical history	**√**								
Inclusion/exclusion	**√**								
Randomization	**√**								
MRI	**√**								
Baseline assessment	**√**								
Motor symptom assessment	**√**		**√**	**√**	**√**	**√**	**√**	**√**	**√**
Quality-of-life assessment	**√**		**√**	**√**	**√**	**√**	**√**	**√**	**√**
Video recording	**√**		**√**	**√**	**√**	**√**	**√**	**√**	**√**
PEGR			**√**	**√**	**√**	**√**	**√**	**√**	**√**
Non-motor symptom assessment	**√**					**√**			**√**
Blinded rater evaluation	**√**	**√**	**√**	**√**	**√**	**√**	**√**	**√**	**√**
BoNT injection		**√**							
FEO/CO application			**√**	**√**	**√**	**√**	**√**	**√**	**√**
Clinical assessments	**√**	**√**	**√**	**√**	**√**	**√**	**√**	**√**	**√**
Safety assessment	**√**	**√**	**√**	**√**	**√**	**√**	**√**	**√**	**√**

BoNT, botulinum toxin; CO, coconut oil; FEO, frankincense essential oil; MRI, magnetic resonance imaging; PEGR, Patient Evaluation of Global Response.

Patients will be withdrawn for the follow-up during a 24-week observation period if they meet any of the following three conditions: (1) the subject receives a BoNT-A reinjection during the follow-up period, (2) the JRS score returns to the baseline score, or (3) completion of a 24-week follow-up visit.

### 2.10 Statistical analysis

Trial analysis will be performed on an intention-to-treat (ITT) population. No interim analyses will be performed. A full statistical analysis plan will be developed before the final analysis.

Assumptions for parametric tests will be evaluated before analysis. Normality will be assessed using the Shapiro–Wilk test and Q-Q plots, and homogeneity of variances with Levene’s test. If these assumptions are not met, non-parametric alternatives will be used.

Missing data will be handled under the ITT principle, using all available observations without imputation beyond the last completed visit. Mixed-effects models will be used for repeated-measures secondary outcomes under the missing-at-random assumption. A per-protocol sensitivity analysis will also be conducted.

#### 2.10.1 Baseline demographic characteristics

Age, educational level, MMSE, HAMA, and HAMD scores will be expressed as mean and SD for normally distributed variables or median and interquartile range (IQR) for non-normal continuous variables. Differences between the two groups will be compared using the *t*-test or Mann–Whitney U-test, as appropriate. Sex will be expressed as frequencies and percentages, and the sex distribution of the participants will be compared using the Pearson *χ*^2^ test or Fisher’s exact test.

#### 2.10.2 Primary endpoint analysis

The primary outcome metric of the study, which is the duration of effective symptom relief as measured by the JRS severity subscale, will be analysed using a *t*-test or Mann–Whitney U-test to compare the differences between the two groups. The difference and its 95% confidence interval (CI) are also reported.

#### 2.10.3 Secondary endpoint analysis

Secondary outcome metrics include the interval between BoNT treatments, improvement rates in motor symptoms (JRS, BSRS, and BFMDRS), quality of life (BSDI, SF-36, and CDQ-24), and subjective efficacy evaluations (PEGR) at different time points. The interval between BoNT-A treatments will be expressed as mean ± SD with differences analyzed using the two-sample t-test if the data follows a normal distribution; otherwise, median and interquartile range will be used, and the Mann–Whitney U-test will be applied. Improvement rates in motor symptoms, quality of life scores, and subjective efficacy evaluations will be expressed as mean ± SD or median and IQR, depending on the normality distribution. Differences between the two groups and their 95% CI will be reported. Outcomes with repeated-measures continuous data will be compared between the groups using repeated-measures ANOVA. Multiple tests will be corrected using Bonferroni correction. All statistical analyses will be performed using SPSS v.25.0 (IBM Corp). *p* < 0.05 will indicate a statistically significant difference.

### 2.11 Data safety monitoring plan

Data collection during each study visit and patient safety will be monitored by the principal investigator. A multidisciplinary committee consisting of at least two health professionals will be established to conduct a full review of the study details to determine whether a possible harm to the subject’s safety or any additional harm can be extracted during the study.

## 3 Discussion

This study will examine whether adding FEO to BoNT therapy can prolong symptom relief. It will also assess whether this combination improves quality of life compared to BoNT alone. In addition, the safety of topical FEO and CO will be evaluated. Sixty-four subjects will be randomized into two groups, with assessments conducted at multiple time points during a 24-week follow-up period. Outcomes will include the duration of symptom improvement, quality of life, and self-rating of response to efficacy, providing evidence of whether FEO therapy is an effective adjunct therapy for the control of BSP symptoms.

However, certain practical issues require clarification. Because FEO has a strong and characteristic odor that cannot be masked even when diluted or sealed, an open-label design was necessary (39). To minimize subjective bias, all assessments will be conducted by two independent blinded evaluators using the same evaluation protocol. Because pure FEO causes irritation when applied directly to the skin, it must be diluted 1:1 with a carrier oil such as CO (16). In this study, CO was chosen as the diluent, but its potential effects on BSP are unknown. Therefore, to control for any influence of CO itself, the control group will receive topical CO alone, applied using the same dosage and massage procedure as in the experimental group.

This study focuses on patients with BSP experiencing shorter relief periods after BoNT treatment, and thus require more frequent injections. Increasing the frequency of BoNT injections leads to higher costs, more side effects, and a greater risk of antibody production, which may result in BoNT resistance and reduced efficacy of subsequent BoNT treatments (40, 41). To address the issue of limited efficacy duration of pretarsal injections in some patients with BSP, Hu et al. (42). Employed a modified BoNT injection method (combining pretarsal with preseptal injections) and compared the efficacy of this method with the pretarsal injections. The study found that pretarsal-preseptal injections achieved a longer duration of response [135.00 (118.50, 153.75) vs. 121.00 (107.00, 135.00) days] than pretarsal injections. In addition, Shukla et al. (8) found that a combined treatment of rTMS to the ACC with a double-cone coil and BoNT could significantly improve motor symptoms 2 weeks after the completion of rTMS treatment compared to BoNT alone. However, the duration of increased efficacy of BoNT injections was limited in both studies. A recent case report showed that topical FEO could prolong the interval between BoNT injections and achieve significant symptom relief in patients with BSP (16). Furthermore, FEO has been proposed as an anti-inflammatory and antioxidant agent that protects neurons and improves motor symptoms in Parkinson’s disease (18). However, whether topical FEO can serve as an effective, low-cost adjunct treatment to significantly increase BoNT remains unknown. Therefore, the primary outcome of the current study is the duration of effective symptom relief within the 24-week observation period, which is defined as a decrease in the JRS severity subscale score by one point or more relative to the baseline (24). If symptom relief is significantly prolonged in the experimental group, this study will provide evidence supporting FEO as an adjunct treatment to potentiate and prolong the effects of BoNT in BSP.

Despite these promising directions, this study has some limitations. In the experimental group, FEO diluted with CO at a 1:1 ratio will be used. Further research should test different concentrations (1:2, 1:3, or 1:5) to determine the optimal concentration associated with positive effects. Second, although FEO may relieve BSP symptoms, further research is required to clarify its potential mechanisms. Third, some patients with BSP experience spontaneous remission, which could affect the results; however, predicting these cases is challenging. Finally, the sensory effects of eyelid massages may contribute to symptom relief. To reduce this bias, the same massage method will be used in the control group.

In conclusion, we propose a study protocol for an open-label, randomized controlled trial to investigate the efficacy and safety of a combination of FEO and BoNT injections in patients with BSP. As a low-cost, non-invasive and easily applicable approach, FEO has the potential to serve as a practical adjunct that may prolong the therapeutic effects of BoNT, and improve symptom control in patients experiencing suboptimal benefit. This trial may therefore provide clinically meaningful evidence to inform a cost-effective and accessible management option for BSP.

## Footnotes

1
https://www.aromatics.com

## Glossary

ACC: anterior cingulate cortex
BFMDRS: Burke Fahn Marsden’s Dystonia Rating Scales
BoNT: botulinum toxin
BoNT-A: botulinum toxin type A
BSDI: Blepharospasm Disability Index
BSP: blepharospasm
BSRS: Blepharospasm Severity Rating Scale
CDQ-24: Cranio-cervical Dystonia Questionnaire
CI: confidence interval
CO: coconut oil
FEO: frankincense essential oil
HAMA: Hamilton Rating Scale for Anxiety
HAMD: Hamilton Rating Scale for Depression
IQR: interquartile range
ITT: intention-to-treat
JRS: Jankovic Rating Scale
MMSE: Mini-Mental State Examination
MRI: magnetic resonance imaging
NIBS: non-invasive brain stimulation
PEGR: Patient Evaluation of Global Response
PSQI: Pittsburgh Sleep Quality Index
rTMS: repetitive transcranial magnetic stimulation
SD: standard deviation
SF-36: Short Form-36 Health Survey
Y-BOCS: Yale-Brown Obsessive-Compulsive Scale
